# Exome sequencing identifies a mutation in the *ACTN2* gene in a family with idiopathic ventricular fibrillation, left ventricular noncompaction, and sudden death

**DOI:** 10.1186/s12881-014-0099-0

**Published:** 2014-09-16

**Authors:** Richard D Bagnall, Laura K Molloy, Jonathan M Kalman, Christopher Semsarian

**Affiliations:** Agnes Ginges Centre for Molecular Cardiology, Centenary Institute, Locked Bag 6, Newtown, Sydney, NSW 2042 Australia; Faculty of Medicine, University of Sydney, Sydney, NSW Australia; Department of Medical Genomics, Royal Prince Alfred Hospital, Sydney, NSW Australia; Department of Cardiology, The Royal Melbourne Hospital, Parkville, Melbourne, Victoria Australia; Department of Cardiology, Royal Prince Alfred Hospital, Sydney, NSW Australia

**Keywords:** Phenotype heterogeneity, Arrhythmia, Cardiomyopathy, Exome sequencing

## Abstract

**Background:**

Potentially lethal and heritable cardiomyopathies and cardiac channelopathies are caused by heterogeneous autosomal dominant mutations in over 50 distinct genes, and multiple genes are responsible for a given disease. Clinical genetic tests are available for several of the inherited cardiac diseases and clinical investigations guide which test to order. This study describes a family with cardiac disease in which marked clinical diversity exists. In the absence of a unified clinical diagnosis, we used exome sequencing to identify a causal mutation.

**Methods:**

Clinical evaluation of family members was performed, including physical examination, electrocardiography, 2D transthoracic echocardiography and review of autopsy records. Exome sequencing was performed on a clinically affected individual and co-segregation studies and haplotype analysis were performed to further confirm pathogenicity.

**Results:**

Clinically affected members showed marked cardiac phenotype heterogeneity. While some individuals were asymptomatic, other presentations included left ventricular non-compaction, a resuscitated cardiac arrest due to idiopathic ventricular fibrillation, dilated cardiomyopathy, and sudden unexplained death. Whole exome sequencing identified an Ala119Thr mutation in the alpha-actinin-2 (*ACTN2*) gene that segregated with disease. Haplotype analysis showed that this mutation segregated with an identical haplotype in a second, previously described family with clinically diverse cardiac disease, and is likely inherited from a common ancestor.

**Conclusions:**

Mutations in the *ACTN2* gene can be responsible for marked cardiac phenotype heterogeneity in families. The diverse mechanistic roles of ACTN2 in the cardiac Z-disc may explain this heterogeneous clinical presentation. Exome sequencing is a useful adjunct to cardiac genetic testing in families with mixed clinical presentations.

**Electronic supplementary material:**

The online version of this article (doi:10.1186/s12881-014-0099-0) contains supplementary material, which is available to authorized users.

## Background

Concerted effort over the past two decades has identified over 50 distinct genes that cause potentially lethal and heritable cardiomyopathies and primary arrhythmogenic disorders. For most of these disorders, there are multiple disease-associated genes [1,2]. Furthermore, mutations in a single gene can cause different diseases, e.g. mutations in the myosin heavy chain 7 *(MYH7)* gene cause hypertrophic cardiomyopathy (HCM), restrictive cardiomyopathy and dilated cardiomyopathy (DCM) [3-5], while mutations in the sodium channel, type V, alpha subunit *(SCN5A)* gene cause long QT syndrome (LQTS) and Brugada syndrome [6,7]. Even within families harbouring the same mutation, incomplete penetrance, phenotype heterogeneity and a variable risk of adverse outcomes are common. In spite of the low genotype-phenotype correlation in the cardiomyopathies and cardiac channelopathies, recent guidelines and expert opinion recommend genetic testing on index cases with a sound clinical suspicion for disease [8], as a genetic diagnosis can explain why the disease has occurred, and allows predictive testing of other asymptomatic at-risk family members.

Commercial genetic tests are available for several of the inherited cardiac diseases, and clinical evaluations guide which test to order. Typically, only the genes most frequently associated with a given disease are screened for mutations. However, advances in targeted massively parallel sequencing, or next-generation sequencing, have overcome the limitations of traditional direct DNA sequencing, and ever-expanding panels of genes are available for screening, including all of the protein-coding genes, i.e. the exome [9,10],

This study describes the clinical and genetic investigations of an Australian family with marked cardiac phenotype heterogeneity among four individuals, including left ventricular non-compaction (LVNC), idiopathic ventricular fibrillation, DCM and sudden unexplained death, which lent itself to the application of exome sequencing to identify a pathogenic mutation.

## Methods

### Clinical evaluation of family members

Clinical evaluation of family members was performed at the Genetic Heart Diseases Clinic at Royal Prince Alfred Hospital, Sydney, and Melbourne Heart Centre, The Royal Melbourne Hospital, Parkville, Australia, with written informed consent and in accordance with the local human ethics standards. Clinical evaluation included detailed personal and family histories, physical examination, transthoracic echocardiography, 12-lead electrocardiogram (ECG) recording, cardiac magnetic resonance imaging (MRI), 24-hour Holter monitoring, exercise testing and review of medical records and autopsy reports.

### Exome sequencing

Genomic DNA was extracted from buffy coat using a QIAmp DNA blood mini kit (Qiagen, Limburg, NL), according to manufacturer’s recommendations. An adaptor-ligated DNA sequencing library was prepared, enriched for the Illumina TruSeq Exome, and paired-end sequenced using an Illumina HiSeq2000 (Macrogen Facility, Seoul, Korea). The 101 bp sequences were aligned to the human genome sequence (GRCh37/hg19) using BWA v0.7.4 [11] with the default parameters. Sequence alignment files were sorted, converted to binary format and indexed using SamTools v0.1.19 [12], and duplicate reads were removed using Picard tools v1.81 (http://picard.sourceforge.net/index.shtml). The Genome Analysis Tool Kit v2.7.2 [13] (GATK) was used for read realignment around insertions/deletions, base quality score recalibration and genotyping of simple nucleotide variations (SNVs) and short insertions and deletions (InDels) using UnifiedGenotyper, according to the GATK best practices (http://www.broadinstitute.org/gatk/guide/best-practices). SNVs and InDels were annotated using SeattleSeq Annotation tool v8.07 (http://snp.gs.washington.edu/SeattleSeqAnnotation137/index.jsp) and compared against the November 2010 release of the 1000 Genomes Project data (http://www.1000genomes.org/), the National Heart, Lung and Blood Institute Exome Sequencing Project (ESP) data (http://evs.gs.washington.edu/evs_bulk_data/ESP6500SI-V2-SSA137.GRCh38-liftover.snps_indels.txt.tar.gz) and in-house exome sequences of 96 unrelated individuals using custom Perl scripts. Estimates of gene expression levels (FKPM) in cardiac tissue were determined with a custom built RNASeq analysis pipeline using data from Illumina’s Human BodyMap 2.0 project (ftp://ftp.sra.ebi.ac.uk/vol1/fastq/ERR030/ERR030886/ERR030886_1.fastq.gz; ftp://ftp.sra.ebi.ac.uk/vol1/fastq/ERR030/ERR030886/ERR030886_2.fastq.gz).

### Variant validation using Sanger sequencing

For validation of detected variants, genomic regions up to 500 bp surrounding variants of interest were PCR amplified, excess primers and deoxynucleotide triphosphates removed using alkaline phosphatase (New England Biolabs, MA, USA) and exonuclease I (New England Biolabs), respectively, and Sanger DNA sequenced (Macrogen). Sequencing electropherograms were manually inspected using Sequencher v5.1 (Gene Codes Corp, MI, USA).

### Haplotype analysis

Primer sequences were designed, with a 6-FAM fluorophore on the reverse primer, to PCR amplify a variable number of tandem repeat (VNTR) polymorphism in alpha-actinin 2 (*ACTN2*), and ryanodine receptor 2 (*RYR2*), plus markers D1S2670, D1S285 and D1S2678 (Additional file 1). PCR was performed at 95°C for 2 minutes, followed by 30 cycles of 95°C for 30 seconds, 54°C for 30 seconds and 72°C for 30 seconds, followed by a final extension at 72°C for 5 minutes. PCR amplicons were separated on an ABI 3730xl DNA analyser (Macrogen) with GeneScan™ 500 LIZ™ size standard (Life Technologies, CA, USA) and alleles sized using Peak Scanner^TM^ software v1.0 (Life Technologies).

All studies were conducted with strict approval and in accordance with the Sydney Local Health District Ethics Review Committee (RPAH zone), Australia.

## Results

### Clinical characterisation of family ALB

**Figure 1:**
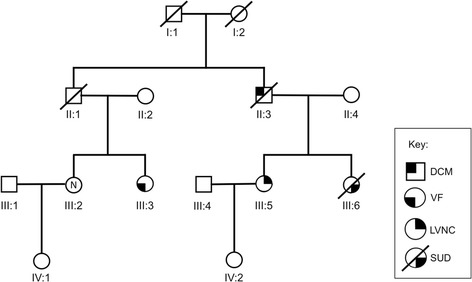
**ALB Family Pedigree. **Squares - males; Circles - females; Line through symbol – deceased individual; open symbol with N – clinically unaffected individual; LVNC - left ventricular non-compaction; VF – ventricular fibrillation; DCM – dilated cardiomyopathy; SUD – sudden unexplained death.

**Figure 2:**
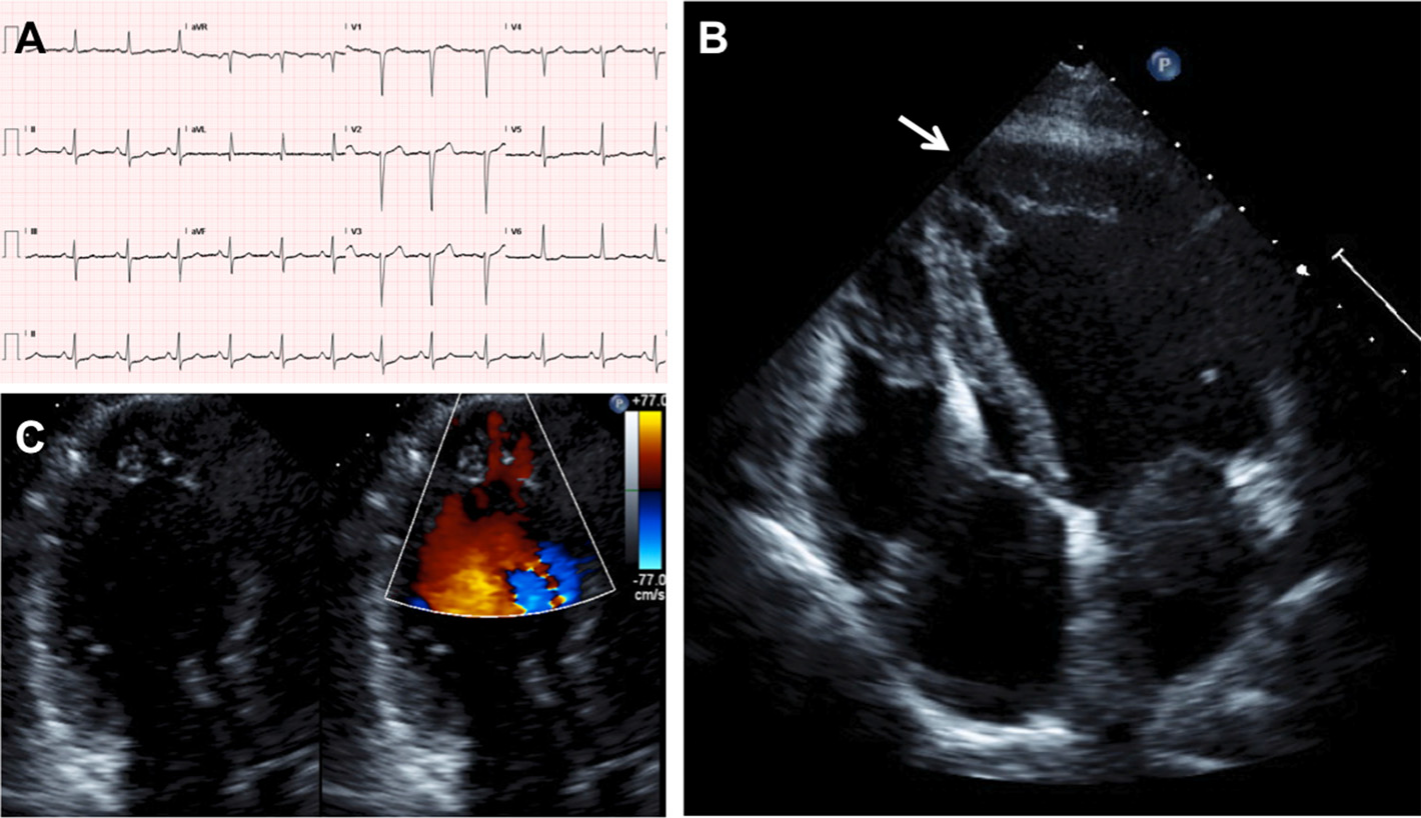
**ECG and echocardiographic features of the proband (III:5).** The ECG **(A)** shows minimal pathological changes, while the echocardiogram shows **(B)** LVNC predominantly affecting the left ventricular apex (arrowed) and **(C)** colour Doppler flow between the trabeculations.

The clinical characteristics of family ALB are summarised in Table 1 and the pedigree illustrated in Figure 1. The family demonstrates marked clinical heterogeneity. The female proband (III:5) presented at age 22 years for clinical screening with a history of syncope and a family history of premature sudden unexplained death. Her sister (III:6) died suddenly during sleep at age 25 years, and her post-mortem failed to identify a cause of death. The proband’s ECG showed sinus rhythm with non-specific ST-T wave changes, whilst her echocardiogram and MRI showed prominent left ventricular apical trabeculations with preserved left ventricular systolic function, consistent with LVNC (Figure 2). There were no inducible arrhythmias during an electrophysiological study, and her QTc measured 440 ms. An implantable cardioverter defibrillator (ICD) was subsequently implanted and she remains stable, with no ICD discharges to date.

**Table 1:** **Clinical characteristics in family ALB**

**ID**	**Gender**	**Current age (yrs)**	**Age at echo (yrs)**	**LVmax (mm)**	**PW (mm)**	**LVEDD (mm)**	**EF (%)**	**ECG**	**ICD**	**Appropriate ICD shocks**	**Final diagnosis**
II-3	M	Deceased	60	12	12	58	27	LBBB	No	NA	DCM
III-2	F	35	29	6	6	51	N	Normal	No	NA	Normal
III-3	F	33	27	6	6	49	N	Minor T-wave changes	Yes	2	IVF (RCA)
III-5	F	35	34	6	4	51	N	ST-T wave changes	Yes	0	LVNC
III-6	F	Died 25	NA	NA	NA	NA	NA	NA	No	NA	SUDY

LVmax - maximum left ventricular wall thickness; PW - posterior wall thickness; LVEDD – left ventricular end-diastolic diameter; EF – ejection fraction; ICD – implantable cardioverter-defibrillator; ECG – electrocardiogram; LBBB – left bundle branch block; DCM – dilated cardiomyopathy; RCA – resuscitated cardiac arrest; IVF – idiopathic ventricular fibrillation; LVNC – left ventricular non-compaction; SUDY – sudden unexplained death in the young; N – normal; NA – not applicable.

**Figure 3:**
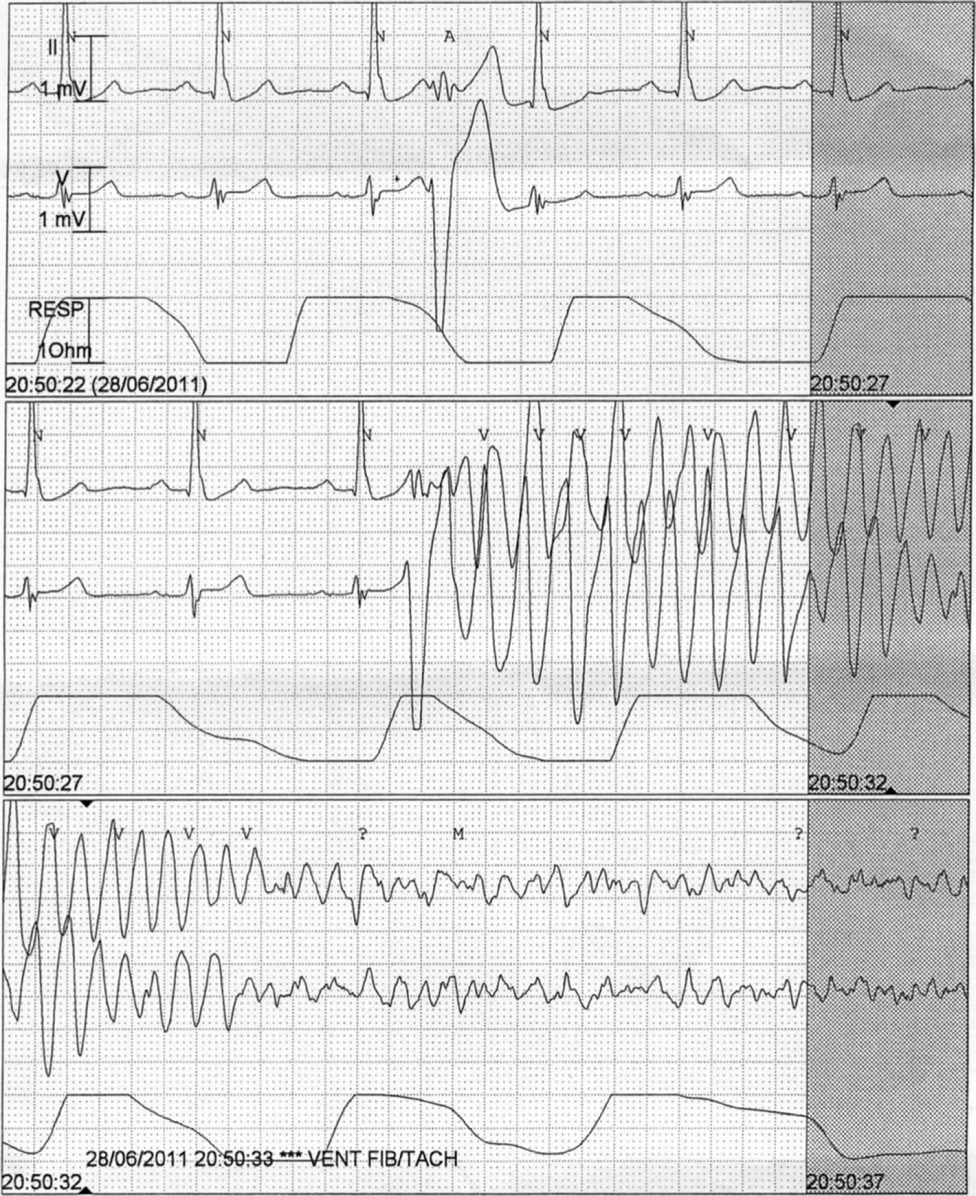
**Ventricular fibrillation in relative III:3.** Example of a short coupled ventricular ectopic triggering VF, as is typically seen in idiopathic ventricular fibrillation.

The proband’s father (II:3) had a history of dyspnoea, left bundle branch block and left ventricular dilatation, with a reduced left ventricular ejection fraction of 27%. The cause of his DCM remained unclear. One female cousin of the proband (III:3) experienced a resuscitated out of hospital cardiac arrest. MRI revealed normal left and right ventricular indexed dimensions and function, with no evidence of myocardial fibrosis, and no features of cardiomyopathy. She was found to have idiopathic ventricular fibrillation and an ICD was implanted, which has subsequently delivered two appropriate shocks (Figure 3). She has responded successfully to quinidine therapy. Her sister (III:2), age 29 years, has been repeatedly evaluated and all cardiovascular investigations have been normal, including a normal ECG, echocardiogram, electrophysiological study and a 7-day Holter monitor. Clinical data, including cardiac screening results, were not available for the proband’s grandparents (I:I and I:2) and uncle (II:1).

### Exome sequencing

To identify a pathogenic mutation responsible for the cardiac phenotype in family ALB, exome sequencing was performed on the proband’s DNA. 62,859,256 sequence reads of 101 bp were generated, of which 38,964,387 mapped uniquely to target regions, representing an average exome-wide coverage of 50.7 reads, with 82.2% of target bases covered at least 20 times. 44,508 SNVs and 4,837 InDels were identified across the 62 Mb target exome, of which 8,214 SNVs caused a non-synonymous, splice-site or nonsense change and 410 InDels occurred in the coding regions or splice signal sequences. We excluded all homozygous variants and heterozygous variants present at >1% frequency in any ethnic subgroup of the 1000 Genomes Project data, or in 6500 exomes of ESP, leaving 259 rare non-synonymous, splice site and nonsense SNVs in 250 genes (Additional file 2) and 13 rare in-frame, frameshift and splice site InDels in 13 genes (Additional file 3). Genes were sorted by expression level in cardiac tissue, derived from RNAseq data, which ranked *ACTN2* Ala119Thr non-synonymous variation as number 1; the top 20 ranked variations are shown in Table 2.

**Table 2:** **Ranked cardiac expressed exome variants identified**

**Chr**	**Nucleotide**	**Ref base**	**Alt base**	**Gene symbol**	**Gene name**	**Cardiac expression (FKPM)**	**Amino acid replacement**	**rs SNP ID**	**ESP6500 alleles**	**1 K Genomes allele frequency**	**GERP score**	**Grantham score**	**PolyPhen score**
**1**	**236882307**	**G**	**A**	**ACTN2**	**Actinin, alpha 2**	**581.019**	**Ala119Thr**	**0**	**G = 13006**	**0**	**5.56**	**58**	**0.999**
**12**	**33049590**	**C**	**T**	**PKP2**	**Plakophilin 2**	**257.408**	**Asp26Asn**	**143004808**	**T = 60/C = 12284**	**0.0027**	**4.07**	**23**	**0.992**
3	49158893	C	T	LAMB2	Laminin, beta 2	241.959	Ala1745Thr	142041381	T = 5/C = 13001	0	5.34	58	1
20	6012009	C	T	CRLS1	Cardiolipin synthase 1	54.3269	Pro119Leu	0	C = 13004	0	5.83	98	0.996
6	138428407	G	A	PERP	TP53 apoptosis effector	50.2039	Ala24Val	0	G = 12832	0	−2.97	64	0
X	32509625	A	C	DMD	Dystrophin	41.8677	Asn789Lys	72468681	C = 63/A = 10498	0.01	0.181	94	0.856
10	81109459	G	C	PPIF	Peptidylprolyl isomerase F	40.9854	Gly89Arg	145899672	A = 3/G = 13003	0	5.4	125	1
2	44200942	T	C	LRPPRC	Leucine-rich pentatricopeptide repeat containing	35.1996	Asn418Ser	0	C = 1/T = 13005	0	2.14	46	0.199
19	11617100	C	T	ECSIT	ECSIT homolog	26.5929	Glu185Lys	0	C = 12994	0	5.4	56	0.999
19	11623977	T	C	ECSIT	ECSIT homolog	26.5929	Asn211Ser	202141211	T = 13006	0	4.94	46	1
**3**	**38645378**	**G**	**T**	**SCN5A**	**Sodium channel, type V, alpha subunit**	**26.2725**	**Ala572Asp**	**36210423**	**T = 21/G = 12443**	**0**	**3.19**	**126**	**0.116**
19	48257869	C	T	GLTSCR2	Glioma tumor suppressor candidate region gene 2	25.0259	Thr284Met	200463741	T = 58/C = 12146	0.0027	1.65	81	0.927
3	33633938	T	C	CLASP2	Cytoplasmic linker associated protein 2	21.537	Lys440Glu	0	T = 11976	0	5.68	56	0.525
9	131341997	T	G	SPTAN1	Spectrin, alpha, non-erythrocytic 1	21.2749	Ser435Ala	144787939	G = 26/T = 12980	0	5.35	99	0.614
2	238280543	C	T	COL6A3	Collagen, type VI, alpha 3	21.1203	Ala1373Thr	112181324	T = 12/C = 12994	0.0018	−0.587	58	0.635
5	122135444	A	G	SNX2	Sorting nexin 2	20.0545	Glu95Gly	141745241	G = 6/A = 13000	0	5.25	98	0
5	57755609	G	C	PLK2	Polo-like kinase 2	19.4331	His60Asp	140600076	C = 14/G = 12940	0	5.26	81	0.008
9	140006432	A	G	DPP7	Dipeptidyl-peptidase 7	18.8162	Met367Thr	78671427	G = 47/A = 12959	0.0018	3.97	81	0.946
2	39509689	T	C	MAP4K3	Mitogen-activated protein kinase 3	18.6804	Ile511Val	78142399	C = 57/T = 12949	0.0014	5.21	29	0.007
6	31600314	G	C	PRRC2A	Proline-rich coiled-coil 2A	18.6379	Glu1288Asp	0	G = 8436	0	−2.26	45	0

NB: Genes associated with a primary cardiac phenotype are shown bold.

### Genotyping and haplotype analysis

Three non-synonymous variants in cardiac disease-associated genes were selected for co-segregation analysis in family members as they affected conserved residues in genes associated with primary cardiac pathologies: Ala119Thr in *ACTN2*, previously reported in a family with heterogeneous HCM [14], Ala572Asp in *SCN5A* (rs36210423), and Asp26Asn in plakophillin 2 *(PKP2)* (rs143004808). Sanger DNA sequencing confirmed the presence of each variant in the proband’s DNA, however, only Ala119Thr in *ACTN2* co-segregated with disease in all of the affected family members, and was also present in one clinically unaffected female (III:2; Figure 4A, B).

**Figure 4:**
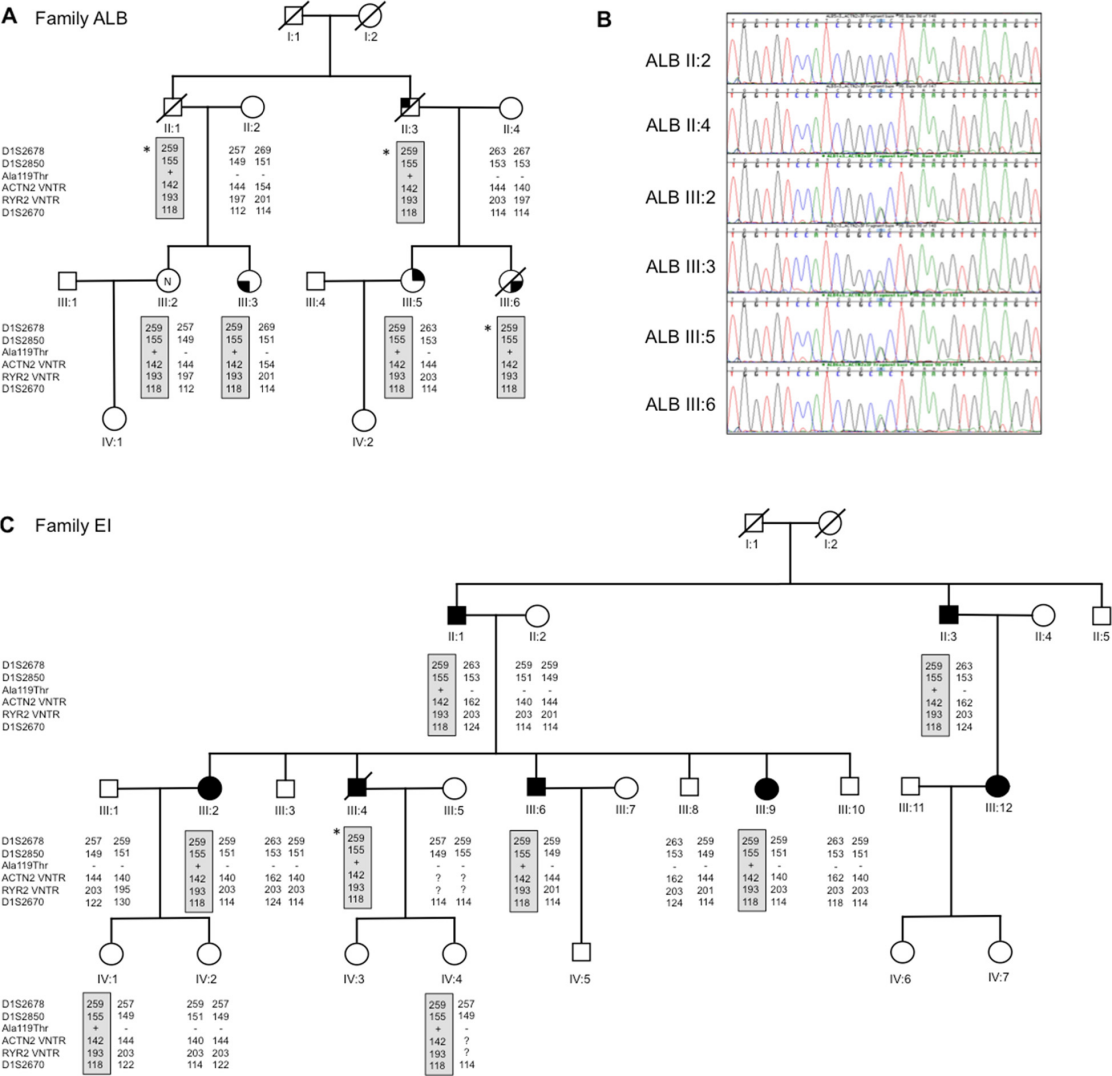
**Genotyping and haplotype analysis in ALB and EI families. (A)** Haplotype analysis in the current Family ALB, **(B)** DNA sequences of the haplotype, and **(C)** haplotype analysis in previous Family EI. *inferred haplotype.

Since we had previously identified the *ACTN2* Ala119Thr variant in an apparently unrelated Australian family with clinically heterogeneous HCM [14], we performed haplotype analysis to determine if the variant segregates on the same haplotype in both families. Five VNTRs spanning a 2.9 Mb region around *ACTN2* were selected; D1S2850 and D1S2670, as used in our previous study, plus one VNTR each in *ACTN2* and *RYR2*, and D1S2678. Haplotype analysis revealed that the Ala119Thr variant segregates with a unique haplotype common to both families, defined by allele sizes of 259 bp in D1S2678, 155 bp in D1S2850, 142 bp in the *ACTN2* VNTR, 193 bp in the *RYR2* VNTR and 118 bp in D1S2670 (Figure 4A, C). We previously determined that the Ala119Thr variant was absent in 297 unrelated probands with HCM, however, since LVNC was a feature in both ALB and EI families, we genotyped a panel of 31 unrelated patients with LVNC. The Ala119Thr variant was absent in all samples (not shown).

## Discussion

Clinical evaluation of an Australian family revealed diverse cardiac pathologies in four affected members and genetic testing of the exome identified a pathogenic *ACTN2* heterozygous variant (Ala119Thr) that co-segregated with disease. The variant affects an amino acid in the highly conserved actin-binding CH1 domain [14]. The diverse clinical phenotypes seen in this family, including idiopathic ventricular fibrillation, LVNC, and sudden unexplained death, suggest mutations in the *ACTN2* gene likely perturb a number of different mechanical and arrhythmogenic substrates. In families with such variable clinical presentations, a non-biased whole exome sequencing approach may be useful in elucidating the underlying genetic cause of disease.

We previously identified the same novel mutation as the cause of a marked clinically heterogeneous cardiac phenotype in a 3 generation family with 27 members (EI family) using genome-wide linkage analysis [14]. The Ala119Thr mutation is absent from 260 control samples, 6500 ESP exomes and the 1000 Genomes data, and segregates with the same unique haplotype in the ALB and EI families. The variant segregates with all affected members of both families (n = 11). Collectively this further validates the pathogenicity of the Ala119Thr mutation in *ACTN2*, which is likely inherited from a common ancestor of both families.

The diversity of clinical phenotypes is a major fascination both in the current (ALB) and our previously reported (EI) families. The spectrum of clinical presentations and clinical outcomes is remarkably similar in both families, and includes both structural and arrhythmogenic pathologies. Several individuals have either hypertrophic cardiomyopathy (both classical and apical forms) or isolated LVNC. Cardiac arrhythmogenic abnormalities, including atrial and ventricular fibrillation, resuscitated cardiac arrest, and appropriate ICD discharges have been reported in a number of individuals in both families. Furthermore, there have been two sudden cardiac deaths in young people aged under 35 years, one where hypertrophic cardiomyopathy was known, and one in which no cause was identified at post-mortem. Heart transplantation for end-stage heart failure has occurred in one individual, presumably due to “burnt-out” hypertrophic cardiomyopathy.

While phenotypic heterogeneity and incomplete penetrance are common features of inherited cardiac diseases, it is unclear how divergent cardiac phenotypes emerge from the same mutation, and so exemplified by the structural and arrhythmogenic pathologies caused by the Ala119Thr mutation in *ACTN2*. The ACTN2 protein primarily functions to anchor and crosslink actin filaments in the cardiac Z-disc at the lateral boundaries of the sarcomere [15]. The Z-disc provides structural support, by tethering the sarcomere to the sarcolemma via the costameres, and by anchoring filamentous F-actin, titin and nebulette [16]. Depletion of *actn2* expression in zebrafish embryos, using antisense oligomers, results in a disruption of the lateral alignment of discs [17]. In humans, analysis of a missense and nonsense mutation in the ACTN2 interacting protein myopalladin [18], revealed a mutation dependent disruption to myofibrillogenesis, or abnormal assembly of terminal Z-discs [19]. Therefore, ACTN2 and the Z-disc can be regarded as a scaffold. Additionally, the Z-disc serves as an interaction platform for proteins that shuttle to the nucleus, such as calcineurin, ankyrin repeat domain 2 and cardiac ankyrin repeat protein, and they may represent molecular messengers that translate mechanical stress into a transcriptional response [20-23]. Therefore the Z-disc can also be regarded as a signalling network that is well positioned to sense mechanical stress [20,24].

**Figure 5:**
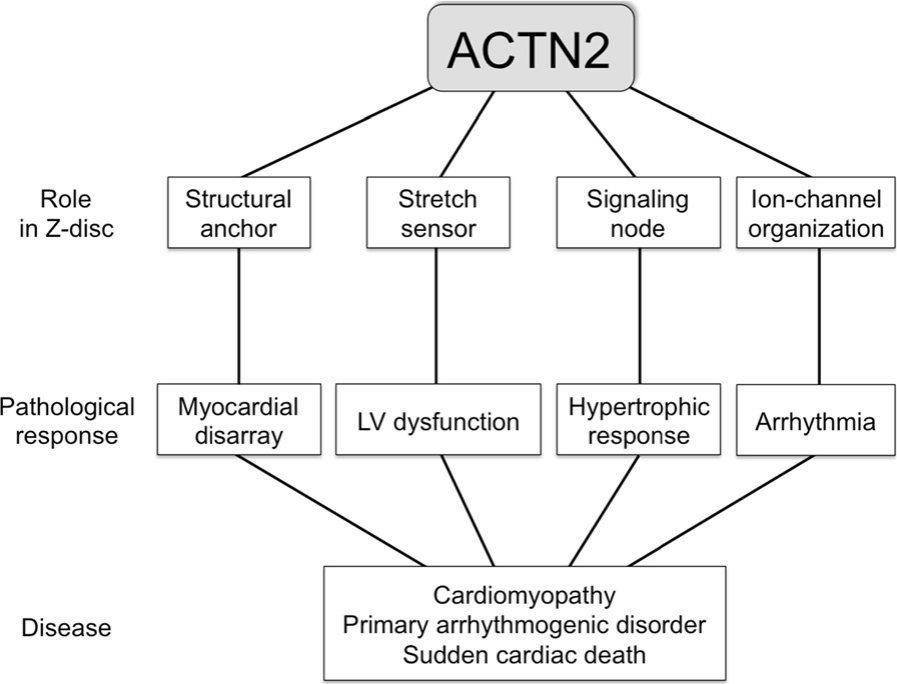
**Alpha-actinin2 and cardiac disease.** Potential mechanisms by which mutations in the *ACTN2* gene can lead to diverse cardiac phenotypes.

Furthermore, there is evidence of ACTN2 directly interacting with cardiac ion channels, such as the potassium ion channels *KCNA4* and *KCNA5* [25,26], the sodium ion channel *SCN5A* [27], and it forms a bridge between the calcium ion channels *CACNA1C* and *CACNA1D* [28]. Thus, disruption of ACTN2 may impact on the localisation and function of cardiac ion-channels. It is tempting to speculate that the different clinical presentations of Ala119Thr result from a stochastic disruption to one of the many functional roles of ACTN2 (Figure 5).

The precedent of mutations in a single gene leading to diverse clinical phenotypes has been documented in other settings. Most notably, mutations in the lamin A/C (*LMNA*) gene have been identified in a diverse range of clinical phenotypes. Originally described in Emery Dreifuss muscular dystrophy, mutations in the lamin A/C gene have now been identified in a number of other diverse diseases including familial dilated cardiomyopathies with conduction disease, limb girdle muscular dystrophies, lipodystrophies, Hutchinson-Gilford progeria syndrome, and mandibuloacral dysplasia, collectively called the “laminopathies” [29,30]. Like the multifaceted role of lamins, and their impact on various biological signalling pathways and functions, the ACTN2 protein has similar diversity in its potential mechanistic roles (Figure 5), and may contribute to a collection of these diseases as “Z-discopathies”.

The current study highlights the emerging role of exome sequencing in the identification of a causative gene mutation. The traditional approach of using the clinical phenotype to select the appropriative genetic testing panel would have required consideration of a cardiomyopathy, sudden death, or arrhythmogenic gene panel, based on the diverse phenotype of the family. *ACTN2* is unlikely to be included on an arrhythmogenic gene diagnostic panel or a common cardiomyopathy gene panel in which each gene is responsible for >5% of disease. However, *ACTN2* may be included on comprehensive cardiomyopathy gene panel that includes minor disease-associated genes. Exome sequencing to investigate the genetic basis of disease in this family was non-biased, robust, and comprehensive, and provided the broadest coverage of possible disease genes to facilitate the identification of the causative mutation. A number of studies have recently emerged demonstrating the use of exome sequencing in genetic discoveries in both primary arrhythmogenic disorders and inherited cardiomyopathies, and therefore provides an exciting new approach to disease gene discovery in cardiovascular disease [31-34].

## Conclusions

Cardiac phenotype heterogeneity is a clinical challenge within families with inherited heart disease. Understanding the underlying genetic causes has major implications both in the diagnosis and screening of at-risk family members, and in shedding light on the mechanisms that underpin disease pathogenesis. The mutation identified in the *ACTN2* gene is likely ancestral to both ALB and EI families and is responsible for the marked cardiac phenotype heterogeneity observed in family members, including both structural and arrhythmogenic abnormalities, including sudden death. The diverse mechanistic roles of ACTN2 in the cardiac Z-disc may explain this heterogeneous clinical presentation. The newer approach of exome sequencing is a useful adjunct to cardiac genetic testing in families with mixed clinical presentations.

## Additional files

Additional file 1: Table S1. Primer sequences, and PCR amplification conditions for microsatellite markers. Additional file 2: Table S2. Rare single nucleotide missense, splice site and nonsense variants. Additional file 3: Table S3. Rare insertions and deletions.

## Abbreviations

BrS: Brugada syndrome
DCM: Dilated cardiomyopathy
HCM: Hypertrophic cardiomyopathy
LQTS: Long QT syndrome
LVNC: Left ventricular non-compaction
SQTS: Short QT syndrome
IVF: Idiopathic ventricular fibrillation
SNV: Single nucleotide variation
ICD: Implantable cardioverter defibrillator
